# Scaling Disturbance Instead of Richness to Better Understand Anthropogenic Impacts on Biodiversity

**DOI:** 10.1371/journal.pone.0125579

**Published:** 2015-05-07

**Authors:** Stephen J. Mayor, James F. Cahill, Fangliang He, Stan Boutin

**Affiliations:** 1 Department of Biological Science, University of Alberta, Edmonton, Alberta, Canada; 2 Department of Renewable Resources, University of Alberta, Edmonton, Alberta, Canada; Technion—Israel Institute of Technology, ISRAEL

## Abstract

A primary impediment to understanding how species diversity and anthropogenic disturbance are related is that both diversity and disturbance can depend on the scales at which they are sampled. While the scale dependence of diversity estimation has received substantial attention, the scale dependence of disturbance estimation has been essentially overlooked. Here, we break from conventional examination of the diversity-disturbance relationship by holding the area over which species richness is estimated constant and instead manipulating the area over which human disturbance is measured. In the boreal forest ecoregion of Alberta, Canada, we test the dependence of species richness on disturbance scale, the scale-dependence of the intermediate disturbance hypothesis, and the consistency of these patterns in native versus exotic species and among human disturbance types. We related field observed species richness in 1 ha surveys of 372 boreal vascular plant communities to remotely sensed measures of human disturbance extent at two survey scales: local (1 ha) and landscape (18 km^2^). Supporting the intermediate disturbance hypothesis, species richness-disturbance relationships were quadratic at both local and landscape scales of disturbance measurement. This suggests the shape of richness-disturbance relationships is independent of the scale at which disturbance is assessed, despite that local diversity is influenced by disturbance at different scales by different mechanisms, such as direct removal of individuals (local) or indirect alteration of propagule supply (landscape). By contrast, predictions of species richness did depend on scale of disturbance measurement: with high local disturbance richness was double that under high landscape disturbance.

## Introduction

The ongoing decline of global biodiversity must be met with a greater understanding of how species diversity and human disturbance are related. A primary impediment to this understanding is that assessments of both diversity and disturbance can depend on scale. Neither diversity nor disturbance is uniformly or randomly distributed in space, so scale dependency of both these variables are predictable features of most systems [1,2]. The concept of scale is thus a cornerstone in many areas of biodiversity theory and conservation (Table 1).

**Table 1:** Concepts of ‘scale’ in biodiversity research and conservation.

Pattern, process, or idea	Use of ‘scale’ concept	Implication for conservation	Key references
Species-Area Relationship	Area of richness estimation	Larger areas harbour more species	[9,83,84]
Island Biogeography Theory	Area of island, distance to mainland	Larger islands, closer to immigration source, harbour more species	[15,85,86]
Habitat loss and fragmentation	Area and insularity of remaining habitat	Larger and more connected patches harbour more species, landscapes with larger, more connected patches harbour more species	[16]
Extinction debt	Area and insularity of remaining habitat	Decline in species delayed following habitat loss or fragmentation	[87–89]
Extinction rate estimation	Area of richness estimation (or endemic richness estimation)	Species extinction is inverse of species area relationship (or endemic-area relationship)	[17,90]
Biodiversity hotspots	Area of richness estimation	Areas with high density of species richness should be protected	[19]
Protected area design	Area and connectivity of reserve	More area protected with greater connectivity among areas may protect more species	[20,91,92]
Local-regional relationships	Area of richness estimation at local and regional scales	Saturated communities can be more easily ‘represented’ in a protected area	[61,91,92]
Intermediate disturbance hypothesis	Extent of disturbance	Areas with intermediate disturbance extent, frequency, or intensity harbour more species	[22]
Metapopulation & metacommunity dynamics	Area and insularity of populations or communities in a region	Regions with larger intact habitats and greater connectivity will harbour more species; Areas from which populations or communities are extirpated may be re-established	[36,93]
Richness-disturbance scale relationship	Area of disturbance extent estimation	Areas with intermediate disturbance extent harbour more species, regardless of disturbance scale estimation; More species expected from locally measured disturbance; Richness depends on both local disturbance and regional disturbance in broader landscape	Current study

Scale dependence of species richness has long been recognised and has received extensive attention over many decades [3–9]. Perceptions of the species-area relationship (SAR) have ranged from a “statistical artefact” [10], a “useful tool for exploring other patterns of biological diversity” [11], to a “fundamental pattern of nature” [12] that is “one of community ecology’s few general laws” [13]. The relationships of both species richness and evenness to spatial scale depend on disturbance [14]. The SAR was a central concept incorporated into theories like Island Biogeography (IBT) [15] and in our understanding of processes such as habitat fragmentation [16]. Research on SAR and IBT has been fueled in part by their application to conservation problems. These ideas have formed the foundation of global extinction rate estimation [17,18], biodiversity hotspot identification [19], and protected area design [20,21].

Despite these many applications of the scale dependence of richness to conservation biology, we investigate an overlooked but important scale-dependent pattern unlike those presented in Table 1. The motivation is that investigation of the scale dependence of the diversity-disturbance relationship has been highly skewed to one side of the diversity-disturbance equation: the focus has been on scale dependence of diversity, while the scale dependence of disturbance has been largely overlooked. Investigations have manipulated either the scale at which species richness is measured, or the scales of richness and disturbance are manipulated together. But the scale at which disturbance is measured is rarely manipulated alone, so the potential influence of variation in disturbance across scales on species richness is largely unknown. Here, we control the scale at which we measure species richness while manipulating the scale at which we measure disturbance. We anticipate new insights from this novel perspective on examining the fundamental relationship between richness and disturbance.

**Fig 1:**
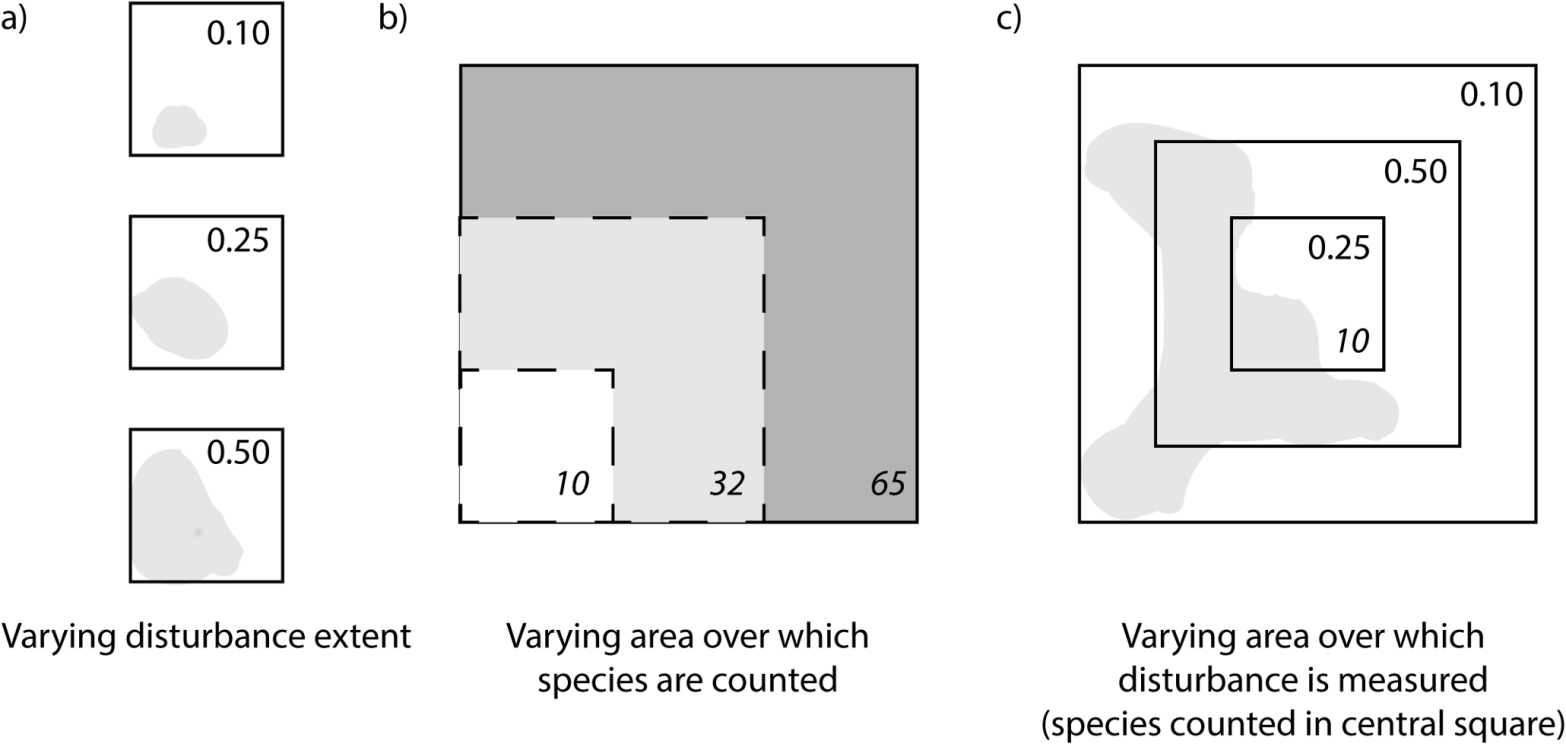
Conceptual approaches to sampling for richness-disturbance relationships. Shading indicates disturbed areas, upper right numbers indicate approximate proportion of area disturbed, and lower right numbers in italics indicate number of species in associated area. In a) the sample area for both disturbance and species richness are identical and do not change, typical of ‘habitat loss’ studies. In b) the sample area over which species are counted varies, a strategy used to estimate the ‘species area relationship’. The largest quadrat is the first sample, in which 65 species were hypothetically found. Dark shading indicates area lost (disturbed) from the first sample, leaving 32 species in the remaining areas. Light shading indicates area lost from the second sample, leaving only the white square area for the third sample, in which 10 species were found. In c) 10 species are counted only in the small central quadrat. Disturbance is first measured in that quadrat, in which a proportion of approximately 0.25 was observed. Disturbance is then measured in multiple larger quadrats, excluding the previous smaller quadrats to minimize dependence of disturbance from one measurement scale to another. This is the sampling approach we followed in the current study, but we measured disturbance at only two scales.

The relationship of diversity to the *scale of disturbance estimation or measurement* (e.g. sample extent, temporal range, range of intensities, etc.) should not be confused with the relationship of diversity to the *scale of disturbance itself* (e.g. spatial extent, duration, frequency, intensity, etc., Fig 1). The latter has garnered much attention, but our focus here is on the former. For instance, among the most prominent theories of the response of richness to disturbance is the intermediate disturbance hypothesis (IDH). The IDH predicts that species richness should show a unimodal relationship to disturbance, such that richness is maximal at moderate extents of disturbance [22]. By contrast, habitat loss and fragmentation studies typically predict declines in richness within remaining patches as disturbance increases [16] and positive richness-disturbance relationships have also been observed [23]. In each of these cases, the possible dependence of species richness on the scale at which disturbance is evaluated is rarely considered. The same is true in many areas of biodiversity research: the measurement scale of disturbance (as opposed to extent of disturbance itself) is neglected or given little attention (Table 1). It is therefore unclear how the richness-disturbance relationship should vary with scales of disturbance measurement. However, because many ecological processes influenced by disturbance vary with scale [24,25], we predict that species richness will be sensitive to disturbance scale. For example, if human disturbance impacts environmental filtering of species, we expect species richness to be related to locally measured disturbance of environmental conditions. But if disturbance alters dispersal or connectivity, we expect disturbance measured in the broader landscape to be related to species richness.

Here, we determine the relationship between species richness of boreal vascular plants and human disturbance extent where disturbance is measured at two spatial scales: local and landscape. Our study provides a test of the IDH at these two disturbance scales. We recently showed that species richness can exhibit a hump shaped relationship to disturbance extent [26], but little is understood of how this relationship may depend on the scale at which disturbance is measured. Following that study, we used samples of richness and human disturbance in 372 sites throughout the 381,047 km^2^ boreal ecoregion of Alberta, Canada. IDH is rarely tested in relation to human disturbances in sites representing such a large region [26,27]. The sites exhibited the full continuum of 0 to 100 percent human disturbance, by area, at both local and landscape scales. They also exhibited a large variety of human disturbance types—differing in intensity, frequency, permanence, and other characteristics—and we evaluate the influence of disturbance types on the results. We tested for two primary patterns: scale dependence in the shape of the richness-disturbance relationship, and scale dependence in model predictions of richness from disturbance. We also tested for those patterns after distinguishing native from exotic species, since these groups have different richness-disturbance relationships’.

## Methods

All necessary permissions were granted by land owners for field surveys. Field studies did not involve endangered or protected species.

Vascular plant richness was surveyed in the boreal ecoregion of Alberta, Canada. The region is approximately 381 047 km^2^ in area. Fine-textured lacustrine and till plains form the dominant landform, and elevations range from approximately 150 m to 1100 m. The region includes deciduous, mixedwood, and coniferous forests and scattered wetlands, but wetlands were excluded from this study. Aspen (*Populus tremuloides*), balsam poplar (*Populus balsamifera*), white spruce (*Picea glauca*), black spruce (*Picea mariana*) and jack pine (*Pinus banksiana) are the primary tree species found [28]. Despite sometimes being characterized as homogeneous, the boreal plant communities vary in composition and structure across the region. The climate is characterized by s*hort summers (only 1 or 2 months have average daily temperatures >15°C) and long, cold winters (average daily temperatures are <−10°C for 4 months or more), with precipitation following a summer-high continental pattern (60–70% of annual precipitation falls between April and August) [28].

We used data and the standardized protocols of Alberta Biodiversity Monitoring Institute [29], and collected supplementary data at additional sites. Vascular plant species occupancy was surveyed within 1 ha for 90 min at a total of 372 sites (see S1 Fig) in an attempt to find all species at each site. We distinguished native and exotic species according to the Alberta Conservation Information Management System [30]. Surveys were conducted between Jun 26 and Aug 18 of 2003 and 2011. Human disturbance extent was defined as proportion of land area converted by humans, and was assessed by manual interpretation of 1:30 000 aerial photos and SPOT satellite imagery at each site by the Alberta Biodiversity Monitoring Institute [31]. For each site, human disturbance extent was measured at two scales: ‘local scale’, the 1 ha square on which plant species were measured; and ‘landscape scale’, a 3 km x 6 km rectangle surrounding the 1 ha site (~ 18 km^2^, [31]). To ensure that the scales represented different information, we selected only these two scales where disturbance was less correlated (*r* = 0.465) because other candidate scales, intermediate to these, were more strongly correlated and analyzing disturbance at those additional scales would have added little information not represented by the landscape and local scales. Disturbance characterized at much larger scales would be unlikely to drive local richness by known mechanisms. The landscape scale plot excluded the central 1 ha, hence the two ‘scales’ were non-overlapping and non-nested. Only the spatial extent of human disturbance was measured, not its intensity, frequency, or time since disturbance, all of which varied greatly within and among disturbance types. Disturbance types were categorized as agricultural (which included pasture and croplands), forestry cut areas (of varying age), hard linear features (permanent and intense; roads and railways), soft linear features (temporary disturbances which allow successional processes; pipelines, powerlines, and cutlines primarily for oil/gas exploration), and urban/industrial (urban and rural settlements, coal and mineral surface mines, oil and gas well pads, communication towers, gravel pits, spoil pads, and heavy oil sands development; [31]).

In interpreting the scale dependence of the species richness-disturbance relationship, we distinguished between two components of the relationship: a) *shape of model*, the difference in regression models (e.g. linear versus quadratic) across local and landscape scales, and b) *predicted richness* of the model along the disturbance gradient, the number of species predicted by the models at a given percentage disturbance extent, with the richness compared between local and landscape scale models. We used 95% confidence ellipses to determine if differences existed between predicted richness across scales.

**Fig 2:**
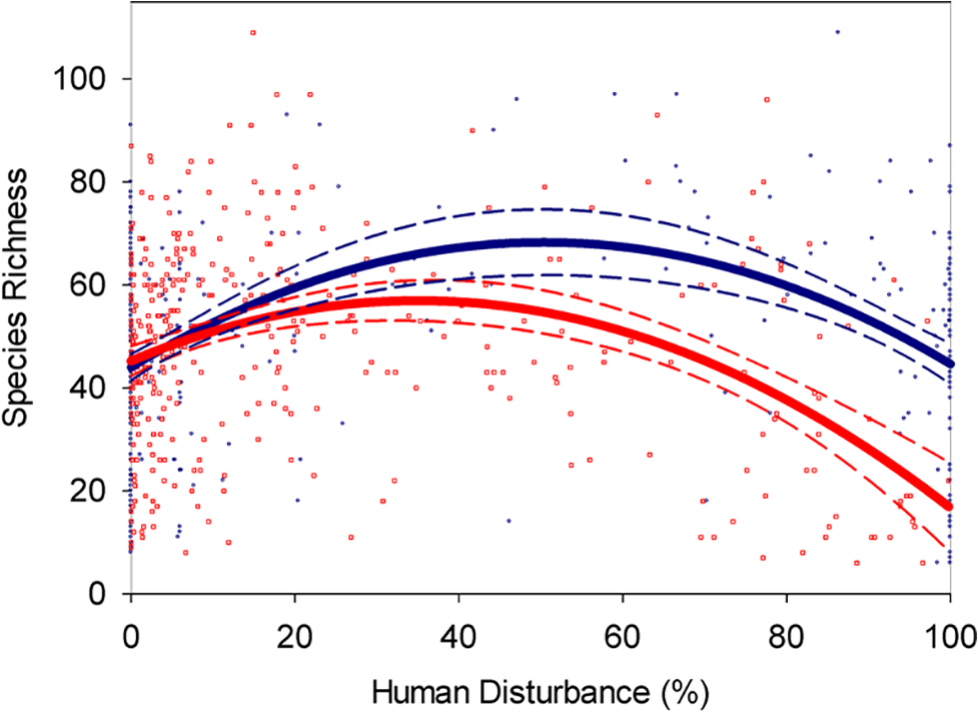
Vascular plant species richness relative to human disturbance extent measured at two scales. Dark blue circles indicate disturbance measured at local scale, red squares at the landscape scale. Corresponding coloured lines are quadratic regression lines of best fit, with dashed lines representing 95% confidence bands.

**Fig 3:**
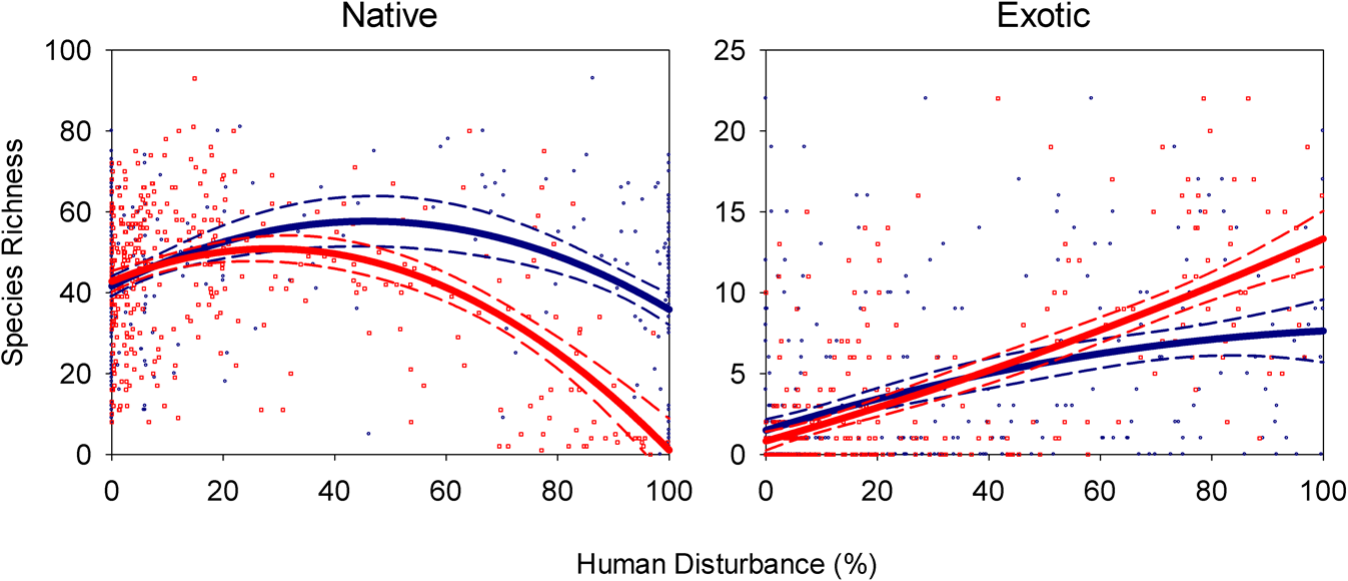
Native and exotic species richness relative to human disturbance extent at two scales. Symbols and lines as in Fig 2.

**Fig 4:**
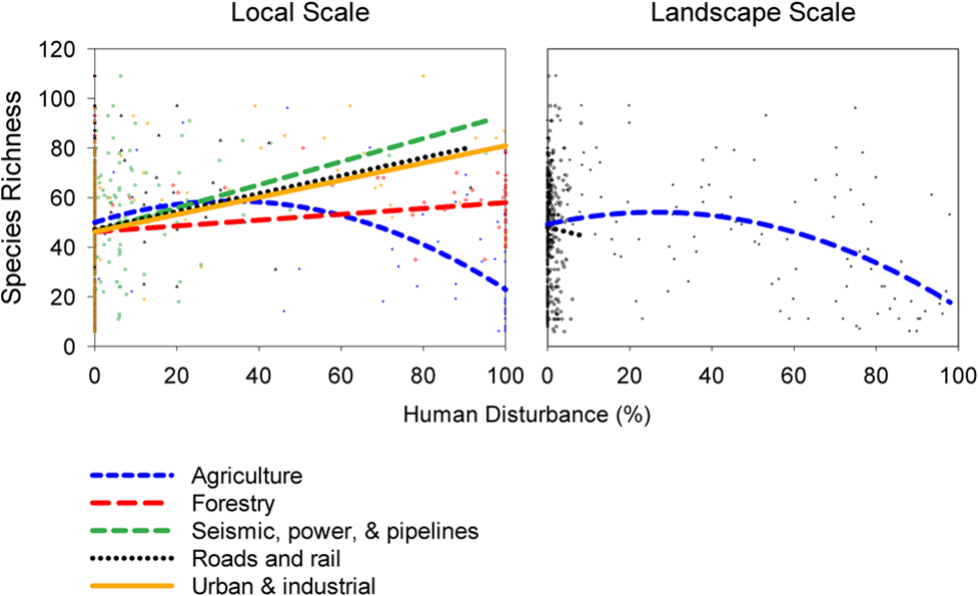
Species richness relative to several types of human disturbance extent, measured at two scales.

To determine the best fit shape of the richness-disturbance relationships, we constructed generalized linear models (GLMs). Statistical analyses were performed in R with package “glm”. Although richness-disturbance relationships are sometimes modeled by simple linear regressions, our GLMs took the form of negative binomial regressions because the species richness data are counts and were overdispersed. Individual GLMs were constructed for local and landscape scales, respectively. We tested GLMs with one (linear), two (quadratic) and three (cubic) parameters. We only adopted higher parameter polynomial models when they were both significant (at *p* < 0.05) and when they fit significantly better (by explaining significantly more variation) than the simpler model of fewer parameters, as diagnosed with an ANOVA of candidate models (reported as “*p* of increase in *R* ^2^ over linear model”). Model selection was conducted according to AIC (highest model likelihood, AIC weight > 0.5). Cubic models were never selected, so aren’t reported. To plot Figs 2–4, we exponentiated the (log-linear) negative binomial models so that they could be plotted on axes comparable to linear models, as they are customarily plotted.

To determine the best fit shape of richness-disturbance relationships while accounting for potentially confounding variables, we constructed GLMs and selected model shapes as above but included additional parameters. For human disturbance and each environmental covariate, we selected the best fitting linear or polynomial form. We used a subset of 192 sites for which data was available for 18 potentially confounding variables. We included in the models human disturbance plus the following: natural subregion type, latitude, longitude, elevation, topographic heterogeneity, growing degree days, mean annual temperature, mean annual precipitation, terrain wetness, site wetness, solar flux, canopy closure, oldest tree age, organic depth, soil type, surficial geology, slope position, and landform classification (see S2 Table). Too few data were available to include natural disturbance (year of last fire and natural disturbance extent) in the models. Details of environmental variable observation and estimation are available at www.abmi.ca.

To determine if richness-disturbance relationships depended on disturbance types, we first determined the best fitting shape richness-disturbance GLM for each disturbance type at the local scale. We then used stepwise selection functions to assess the significance of each disturbance variable. We repeated this procedure for disturbance at the landscape scale. Next, we determined the best multi-scaled model of species richness in relation to disturbance by using stepwise selection functions with each disturbance type at each scale as candidate variables for inclusion.

## Results

In total, 662 different species were observed, including 565 native species and 75 exotic species (and 22 unknown/undetermined). Species richness of boreal vascular plants depended on the extent of human disturbance at two spatial scales (Fig 2, S1 Table). We observed statistically significant relationships (*p* < 0.001) when human disturbance was measured at local and landscape scales. Thus, species richness was not only affected by direct, local disturbance of vegetation, but by distant disturbances in the broader landscape. As expected, human disturbance was correlated across scales (*R* = 0.465), however the consistent dependence of richness on disturbance extent at each scale could not be explained by this relationship. A model including disturbance at both scales explained significantly more variation in richness (*R* ^2^ = 0.226, *p* <0.001) than models of disturbance at either local (*R* ^2^ = 0.108) or landscape scale disturbance (*R* ^2^ = 0.122), respectively.

The shape of the richness-disturbance relationship was also consistent across scales (Fig 2, S1 Table). When linear, quadratic, and cubic models were compared, quadratic relationships best fit species richness relative to disturbance extent regardless of the scale at which disturbance was measured. At each disturbance scale, species richness peaked at intermediate human disturbance extent (Fig 2, S1 Table). The same model was consistently selected by both p-value and AIC in all cases.

Species richness was influenced by many factors besides disturbance, and many of these factors were correlated (S3 Table). When we accounted for these covariates, the percentage total human disturbance extent at the local scale was still significantly related to richness by a quadratic function (*p* = 0.032) which fit significantly better than a linear model (*p* = 0.032, S4 Table). At the landscape scale however, total human disturbance was unrelated to richness (*p* = 0.120). A saturated model with disturbance at both scales and all covariates explained *R* ^2^ = 0.738, only *R* ^2^ = 0.027 more variance than explained by the covariates without disturbance, *R* ^2^ = 0.711 (S4 Table). This situation could result if human disturbances depended on abiotic variables such as latitude. However, we suggest instead that disturbance may also reasonably be expected to alter abiotic environmental variables (see discussion).

Unlike the *shape* of the richness-disturbance relationships, the *number of species* predicted by richness-human disturbance extent relationships depended strongly on the scale at which disturbance was measured (Fig 2). At low to moderate human disturbance (< 45%), species richness predictions were statistically indistinguishable across scales of disturbance. However, at moderate to high disturbance, predicted richness was higher when disturbance was measured at the local scale than at the landscape scale (Fig 2). Species richness was predicted to be 44 species at 100% local disturbance, but only 21 at 100% landscape disturbance, a two-fold difference. Species richness varied most with disturbance extent at the landscape scale, where predicted richness ranged from 21 to 57 species. Predicted species richness ranged from 44 to 68 depending on local disturbance.

Native and exotic species differed in their response to disturbance in several ways. Native species richness was quadratically related to human disturbance extent at all scales (Fig 3, S5 Table). However with landscape disturbance—which explained more variation in native species richness than did local disturbance (*p* < 0.001)—richness decreased significantly more at greater disturbance extent and approached zero species at 100% landscape disturbance. Native species richness peaked at 46.1% local disturbance but only 28.7% landscape disturbance (Fig 3, S5 Table). Native richness predicted by 100% disturbance at the local scale was 35.9 species, very close to the 42.0 native species expected with no disturbance, but with 100% disturbance at the landscape scale only 1.4 species were predicted (Fig 3).

Exotic species richness by comparison showed different shaped richness-disturbance relationships at local and landscape scales (Fig 3, S5 Table). With locally measured disturbance, the relationship was concave down, but with landscape level disturbance the exotic richness-disturbance relationship was concave up (Fig 3). Despite the curvilinear shapes of these relationships, they both tended to increase with disturbance extent and never peaked. Predicted exotic richness at 100% disturbance extent was 6.7 with local disturbance, nearly half the 13.2 exotic species expected with landscape disturbance (S5 Table).

Species richness-disturbance relationships also varied by disturbance type (Fig 4, S6 and S7 Tables). Agricultural disturbance explained the most variation in richness among disturbance types, regardless of whether that disturbance was local or at the landscape scale. Species richness was locally affected significantly by all anthropogenic disturbance types. At the landscape scale, the only anthropogenic disturbance type significantly explaining species richness was agriculture, with the exception of roads and rail, which covered a very small area of the landscape. Although species richness appeared to linearly increase with non-agricultural local disturbances, too few sites exhibited high disturbance extent of these types for the relationship to be considered reliable at high disturbance.

Our complete saturated model, accounting for all environmental variables and considering disturbance types individually, explained more than three quarters of the variation in species richness (*R* ^2^ = 0.768, *p* < 0.001, Table 2). This saturated model, however, contains all the disturbance variables and all the environmental covariates, prior to step selection (where variables which explain little are removed), and so many of the variables may be collinear (see S3 Table) and appear non-significant.

**Table 2:** Saturated model explaining species richness.

Explanatory variable (and scale)	Estimate	df	*r* ^*2*^	*p*	AIC
Forestry (1ha)	0.002102	191	0.768	0.016	1560.10
Hard linear features (1 ha)	0.007002			0.140	
Soft linear features (1 ha)	0.002617			0.287	
Urban and industrial (1 ha)	0.002834			0.078	
Agriculture^2^ (1 ha)	-0.000368			0.008	
Forestry (18 km^2^)	-0.001968			0.614	
Hard linear features (18 km^2^)	-0.005342			0.887	
Soft linear features (18 km^2^)	-0.012560			0.357	
Urban and industrial (18 km^2^)	-0.003674			0.655	
Agriculture^2^ (18 km^2^)	0.000033			0.627	
Natural subregion: Central Mixedwood	0.063080			0.785	
Natural subregion: Dry Mixedwood	-0.069240			0.787	
Natural subregion: Lower Boreal Highlands	0.142000			0.571	
Natural subregion: Northern Mixedwood	0.522100			0.078	
Natural subregion: Peace-Athabasca Delta	0.178300			0.602	
Natural subregion: Upper Boreal Highlands	0.319900			0.280	
Latitude	-0.141000			0.207	
Longitude	0.005969			0.810	
Elevation	0.000184			0.816	
Topography	-0.002132			0.721	
Growing degree days	0.000834			0.498	
Mean annual temperature	-0.033150			0.827	
Mean annual precipitation	-0.002387			0.094	
Terrain Wetness	-0.002379			0.896	
Site Wetness	0.000594			0.404	
Solar Flux	2.242000			0.158	
Canopy cover	-0.003544			0.005	
Tree age	0.000038			0.952	
Organic depth	-0.003374			0.000	
Soil type: Brown Grey Luvisols	0.581000			0.181	
Soil type: Cryosols	0.115700			0.630	
Soil type: Dark Grey Chernozems and Luvisols	0.048210			0.875	
Soil type: Dystric Brunisol	0.135900			0.494	
Soil type: Eutric Brunisols	0.016920			0.962	
Soil type: Gleysols	0.113300			0.592	
Soil type: Grey Solonnetzic Luvisols	0.378800			0.046	
Soil type: Organics	0.271000			0.153	
Soil type: Regosols	0.951400			0.025	
Surficial geology: Eolian	0.639400			0.273	
Surficial geology: Glaciofluvial Complex	1.542000			0.029	
Surficial geology: Glaciofluvial Plain	0.675500			0.244	
Surficial geology: Lacustrine Coarse	0.849200			0.149	
Surficial geology: Lacustrine Fine	0.658200			0.257	
Surficial geology: Organic	0.779100			0.193	
Surficial geology: Till Blanket	0.748100			0.200	
Surficial geology: Till Veneer	0.681100			0.261	
Surficial geology: Water	0.960400			0.136	
Slope position: Midslope	-0.682400			0.113	
Slope position: Toe slope	-0.097950			0.462	
Slope position: Upper slope	0.000862			0.994	
Slope position: Valley	-0.940900			0.197	
Landform class: Mountain ridge top	-0.235700			0.343	
Landform class: Open slope	0.366800			0.434	
Landform class: Plain	-0.322500			0.087	
Landform class: U-shaped valley	-0.188300			0.338	
Landform class: Upper slope	-0.294100			0.143	

Includes all environmental covariates considered (prior to step selection) and the best fit shape of each human disturbance variable (with quadratic variables indicated by “^2^”).

## Discussion

### Local richness depended on disturbance extent at both scales

Our results show that species richness can depend on multiple spatial scales of anthropogenic disturbance. Richness of boreal vascular plants was partly explained by direct local disturbance and disturbance in the broader landscape up to 3 km away. These multi-scaled effects of disturbance on species richness were evident when local and landscape areas of disturbance estimation were non-overlapping, non-nested, and with relatively low correlation in disturbance across scales. Because the two scales at which disturbance was measured offered quantitatively different information, the similarity in shapes of richness-disturbance relationships between scales of disturbance cannot be attributed simply to propagation of patterns at the local scale to the landscape scale or vice versa (*sensu* [32]). Including disturbance at both scales explained nearly double the variation in richness (*R* ^2^ = 0.226) as did either scale individually.

Local human disturbance may alter species richness by a combination of factors: i) direct removal of individuals; ii) direct and indirect alteration of abiotic environmental conditions like sunlight, moisture, and soil characteristics; iii) prevention or inhibition of successional processes by permanent structures or frequent disturbances (e.g. semi-annual tilling, mowing, or brush clearing); iv) indirect alteration of biotic interactions (e.g. herbivory, pollination, competition).

The importance of landscape disturbance outside or surrounding communities or areas of interest has long been recognised and is a hallmark of habitat loss and fragmentation studies. Over 50 years of study have revealed that species richness in habitat patches depends on: i) patch area; ii) edge effects, which alter area of ‘interior’ habitat; iii) biological area effects, such as when patches are too small to support species with large ranges; iv) extirpation cascades, due to impacts on species interactions or extirpation of keystone species; v) patch isolation, due to limitations on dispersal [16,33,34]. Despite these lessons, which often make use of the ‘scale’ concept, habitat loss and fragmentation studies have not yet provided a basis for predicting richness-disturbance relationships across scales. Further, impacts of habitat loss and fragmentation on species richness may also depend on plant community type.

Metacommunity theory [35–37] and its progenitors like island biogeography [15,38,39], fragmentation [16] and source-sink dynamics [40–42], invasion ecology [43–45], and the intermediate disturbance hypothesis [22] help explain conceptually why species richness is affected by disturbance at broader (e.g. landscape) scales. Ecological processes that connect these ideas, like dispersal and isolation, seed rain and propagule supply [46–48], succession [49–52], and the role of environmental heterogeneity [53] and ‘matrix habitat’ may all contribute to observed relationships. Hamer and Hill [14] found that the SAR depended on whether disturbance had occurred or not, and Dumbrell et al [54] suggested that the difference in slope of SARs in disturbed and undisturbed areas drove the scale dependence of the diversity-disturbance relationship.

Human disturbance was correlated with many abiotic environmental variables (S3 Table) and accounting for these variables significantly reduced the proportion of variance explained by disturbance to *R* ^2^ = 0.027 (S4 Table). One might conclude that abiotic variables, not disturbance, drove the observed richness patterns. For instance, variation in environmental variables is well known to contribute to patterns of species richness, irrespective of disturbance. There are several reasons, however, why the effects of disturbance were lower than without accounting for those covariates and why disturbance impacts may be underestimated. First, by accounting for these variables, we effectively assumed that disturbance was driven by those variables. Variables such as latitude, natural subregion, soil type, and climate related variables may influence where humans use land. However, several variables such as canopy cover, depth of organic soil, terrain wetness may instead have been effects of disturbance, so factoring them out could have masked those effects. Second, human disturbance was only considered as a total percentage of any type of disturbance, but disturbance types differed in intensity, frequency, permanence, and other characteristics. Models which included all environmental covariates explained more variability in richness when disturbance types were categorized and treated as individual parameters (*R* ^2^ = 0.768, in the full saturated model) than when disturbance was considered a total (*R* ^2^ = 0.738, S4 Table). Third, because environmental data were unavailable for 176 sites (of 367 sites), we excluded those sites from analyses using environmental covariates.

### Scale invariant shape of richness-disturbance relationships supports intermediate disturbance hypothesis

The richness-disturbance relationships fit quadratic models at each scale of disturbance, suggesting that the unimodal shaped pattern is robust to sampling scale (Fig 2). We also previously found a unimodal richness-disturbance relationship with disturbance measured at a scale between the local and landscape (150 m radius circle; [26]). The consistent shape of the richness disturbance relationships provides strong multi-scale support for the IDH in this system.

Support for IDH across studies has been limited, with approximately 80% of empirical studies failing to support the hypothesis of peaked richness at intermediate disturbance [23,27]. Theoretical support based on a variety of ecological mechanisms has been stronger [27,55], but these models have also been criticized [56]. Shea et al. [27] suggested that the varying support for IDH may be related to study scale, and Hill and Hamer [57] showed that the diversity-disturbance relationship in tropical forest birds depended on spatial scale and not on sampling method. However, Allcock and Hik [58] found that richness-disturbance relationship were similar at two scales following herbivory treatments. Most previous studies of IDH have been conducted at relatively small spatial extents and small sampling grains. We previously showed support for IDH in this system and suggested that the unusually large regional extent of our study area may have contributed to this unusual finding [26]. The consistent support for IDH across scales in this boreal system, but inconsistent findings across other studies conducted at various scales highlight the complexity of scaling biodiversity and disturbance and the limitations of simple models.

### Scales of ecological processes

The cross-scale similarity in shape of richness-disturbance relationships is surprising given our expectation of how local and landscape disturbances would affect richness differently. We expected scale-dependence because ecological processes potentially impacted by disturbance vary in the scales at which they operate. For instance, Garcia and Chacoff [59] showed that different scales of decreases in forest cover drove changes in different functional processes such as pollination, frugivory, and seed predation. Similarly, direct habitat loss has a greater effect on species than does habitat fragmentation per se [16], but the effects of fragmentation increase with proportion of disturbed area [60]. Even the local-regional relationship is dependent on sampling scale: it can appear saturated or not depending on the scale at which it is evaluated [61].

Can the scale at which disturbance is measured in richness-disturbance relationships help elucidate the processes structuring local plant communities? In the current study, dispersal of propagules may occur over very large distances across landscapes, whereas competition is most likely strongest at small, local scales [62]. Coarse scale disturbance in the landscape therefore likely affects processes related to dispersal like external propagule supply and isolation of communities whereas fine scale local disturbance directly removes vegetation and alters environmental and soil conditions, acting as an environmental filter [63,64].

### Scale dependent predictions of richness from disturbance

Despite the qualitative similarity in shapes of richness-disturbance curves, we found contrasting results when these relationships were used to predict species richness. Predictions of species richness from disturbance extent depended strongly on the scale at which disturbance was measured (Fig 2).

More than double the number of species (44) were predicted from 100% local disturbance extent as from 100% landscape disturbance (21 species, Fig 2). The range in predicted species richness with landscape disturbance far exceeded the range predicted with local disturbance. This seems counterintuitive because we expected species richness to be more sensitive to direct, local disturbances of the local vascular plant community than to indirect disturbances surrounding or distant from the local community. The greater sensitivity of richness to landscape scale disturbance could reflect that a given percentage disturbance at that scale represents a much larger disturbed area than the same percentage at the local scale. In addition, only agricultural disturbances (which are intensive and perpetual) were proportionately extensive at the landscape scale, whereas more compact disturbances such as cutblocks or well pads contributed higher percentages of disturbance at the smaller local scale. The varying effects of disturbance types are elaborated upon below.

### Native and exotic species

The relationship of native to exotic richness has often been observed to depend on spatial scale—both extent and grain [65]—with natives and exotics positively correlated at large scales and negatively correlated at smaller scales [66–70]. Davies et al. [71] suggested this scale dependence was a result of spatial heterogeneity, a pattern strongly influenced by disturbance.

Here, native species and exotic species differed dramatically in their responses to disturbance (Fig 3, S5 Table), consistent with past studies [58,72,73]. While native species richness showed a quadratic relationship to human disturbance extent at each scale, supporting the IDH at both scales, exotic richness increased with landscape scale disturbance without peaking, a pattern inconsistent with the IDH. This difference in shape of response of native and exotics to disturbance was consistently observed across scales, but was more pronounced with landscape disturbance.

Predicted richness of each of these two groups both strongly depended on the scale at which disturbance was measured. Native species decreased to only 1.4 species with 100% landscape disturbance while maintaining 35.9 species with 100% local disturbance. Exotics, by contrast, rose from near zero species at 0% disturbance at any scale to 6.7 species at 100% local disturbance or 13.2 species at 100% landscape disturbance. Where disturbance was high at either local or landscape scales, native species declined while exotic species richness increased (Fig 3). Schetter et al. [70] similarly found that richness of natives and exotics was best explained at different scales of land cover. A meta-analysis also showed that the impacts of exotics on species richness declined with the study’s spatial scale [74] and exotic species can also alter species area relationships [75]. Clearly more research is required at the intersection of disturbance, scaling, and exotic species.

### Disturbance Type

The diversity-disturbance relationship can depend on scale and disturbance type simultaneously [76]. Here, disturbance varied dramatically across study sites, both in extent and in type. When we separated the effects of various disturbance types, our models explained significantly more variation in richness (*R* ^2^ = 0.307, Fig 4, S6 and S7 Tables) than when considering the sum extent of disturbance (*R* ^2^ = 0.226). We observed that the disturbance types significantly impacting total species richness across sites depended on the scale at which disturbance was measured.

With landscape disturbance, only agriculture explained richness significantly, apart from some influence of roads and rail lines at < 9% disturbance extent. By contrast, richness was significantly explained by all observed disturbance types when measured locally, including forestry, soft and hard linear disturbances, and urban/industrial disturbance. Even at the local scale, however, most disturbance types only covered a small percentage of the sample areas of most sites, likely driving the linearly increasing, rather than quadratic, patterns of richness-disturbance relationships in Fig 4. It should not be assumed from these data that species richness continually increases with non-agricultural disturbance.

We suggest that the greater decreases in species richness with total landscape disturbance can be at least partially attributed to the intensive nature of agricultural land use relative to other disturbances such as forestry cut blocks or seismic lines, which more typically allow successional processes to take place. Had we measured disturbance at only the local scale, we would have overestimated the impact of temporary disturbances on richness, demonstrating the valuable insight gained by measurement of disturbance at multiple rather than single scales. Further, because agriculture was the only disturbance type at the landscape scale observed at high proportions, our conclusions for impacts of other disturbances on richness must be limited. For example, with high proportions of more intensive disturbances, like heavy oil sands extraction, one might expect greater reductions in species richness, whereas with less intensive disturbances, like forestry, one might expect lesser reductions in richness.

For this study, data describing time since disturbance were unavailable. However, time since disturbance may have been an important axis of disturbance and is well known to influence diversity in the boreal forest [77–79]. This was likely a greater issue at the local scale—where forestry and soft linear disturbances played a greater role and varied on the order of decades—than at the landscape scale, where agriculture played the dominant role and was more consistently recently (months to years) disturbed. Future studies should investigate the scale dependence of time since disturbance on species richness and diversity.

### To what extent was richness explained by disturbance?

Our primary goal in this study was not to explain the maximum amount of richness, it was to explore the scale dependence of impacts of disturbance on richness. Overall, 22.6% of the variation in richness was explained by disturbance alone—not an insignificant amount (S1 Table). However, disturbance is one of many factors explaining disturbance, and our saturated model with environmental variables included was able to explain 76.8% of the variation in richness (Table 2). Further, we report some very low R^2^ values in several instances, such as the variation in exotic richness explained by local disturbance alone (R^2^ = 0.047, S5 Table). Interpretations and conclusions drawn from these low values must be tempered. For instance, one should not conclude that local disturbance alone primarily drove exotic richness in this system. By contrast, our conclusions that exotic species richness rose with disturbance, in stark contrast to the behaviour of native species (which peaked at intermediate disturbance), or that predictions of richness under high disturbance depended on the scale of disturbance estimation, appear to be well grounded.

### Implications for biodiversity management and conservation

One could argue that scales of the effect of disturbance on richness matter little to conservation practitioners because conservation decisions are rarely made on the basis of richness alone. However, Hartley and Kunin [80] report that extinction risk of species and their relative priority for conservation are also affected by scale: biological conservation cannot escape the scale dependence of the biology it aims to conserve.

The current study has several lessons for conservation and management. First, the observed cross-scale impacts of human land use in this study suggest that assessments of environmental impacts or extirpation risks focussing only on direct, local human disturbance likely underestimate the cumulative impacts of disturbance at broader scales.

Second, the use of species-area relationships as predictors of richness relative to disturbance is inadequate. Gains in disturbance may initially seem equivalent to the loss of area, but the two are not equivalent. Disturbance changes environmental conditions rather than removing the environment altogether. Altering the scale of richness and disturbance estimation together may lead to unexpected or nonlinear results because species richness and human disturbance may depend on scale in different ways.

Third, the consistent shape of richness-disturbance relationships and support for the intermediate disturbance hypothesis suggest that in this system, quadratic richness disturbance models can form a simple base level expectation in the absence of more specific information to guide land use planning and management decisions. However the dependence of predicted biodiversity on scale of disturbance estimation suggests predicting the richness-disturbance relationship is more complicated and may best be achieved by multi-scale models.

### Conclusions

The complicated relationship of biodiversity, disturbance, and scale has been explored from many perspectives. Still, Dodds [81] (pg. 168) suggested that a primary impediment to applying the IDH is “how to scale disturbance for effect on communities,” concluding that “we have no *a priori* method of scaling disturbance based on first principles.” Here, we offer field results showing that biodiversity depends on two scales of disturbance with a predictable richness-disturbance shape. However, our results suggest that richness-disturbance parameters depend on the scale at which they are measured. Just as there is no ‘correct’ scale at which to measure species richness [24], there is no single ‘correct’ scale to explore how disturbance affects richness. For example, Huston [82] argued that local processes determine observed regional patterns in diversity, but we show here that landscape scale disturbance affects local diversity independent of local disturbance. The scale dependence of the richness-disturbance relationship is not simply a problem of ‘scaling up’ richness from sample areas to regions. Because human disturbance influences species richness at multiple scales—including scales much larger than those at which richness is measured—the seemingly arbitrary choice of scale at which to measure disturbance may determine expected values of richness.

We aimed in this study to expand our understanding of the richness-disturbance relationship beyond what could be learned from application of the SAR. An important feature of the SAR is that species richness depends not only on sample area, but that the slope of the SAR itself depends on scale; it varies from local to regional, to continental scales [83]. Similarly, our results suggest species richness depends on both disturbed area and on the scale over which disturbed area is measured. When scaling biodiversity to better understand impacts of human land use change, predictions may be aided by considering scales of both diversity and land use change.

## Supporting Information

S1 Fig Map of sample locations in the boreal ecoregion of Alberta. Inset map shows region within Canada, with boreal ecoregion shaded.
(DOCX)

S1 Table Comparison of model shapes of species richness-human disturbance relationships at local (1 ha) and landscape (18 km^2^) scales. (DOCX)

S2 Table Explanatory variables included in this study. All measures observed or estimated at site centre or within entire local 1 ha site, unless otherwise noted. Details are available from the Alberta Biodiversity Monitoring Institute at www.abmi.ca.
(DOCX)

S3 Table Correlation matrix of human disturbance and continuous environmental covariates. Numbers below the diagonal indicate the correlation. Bold values indicate adjusted p < 0.05. Numbers above the diagonal indicate the adjusted p-value.
(DOCX)

S4 Table Species richness-human disturbance relationships at local and landscape scales, accounting for co-variates. The negative binomial regression models were constructed using all parameters (saturated) to facilitate comparisons across models.
(DOCX)

S5 Table Comparison of models of richness-disturbance relationships for native and exotic species. (DOCX)

S6 Table Best models (stepwise selected) explaining richness with types of human disturbance at each scale individually. Only the disturbance types and forms selected by the model are shown. Linear and quadratic forms of each disturbance variable were options, and quadratic variables are indicated by “^2^”.
(DOCX)

S7 Table Best multi-scale model of richness explained by human disturbance types at local and landscape scales. Only the disturbance types, their scales, and the variable forms selected by the model are shown. Linear and quadratic forms of each disturbance variable at each scale were options, and quadratic variables are indicated by “2”.
(DOCX)
