# Supplementary material for: Scaling Disturbance Instead of Richness to Better Understand Anthropogenic Impacts on Biodiversity
Source: PLoS One. 2015 May 7;10(5):e0125579. doi: 10.1371/journal.pone.0125579 (PMC4423832; doi:10.1371/journal.pone.0125579)

Fig. S1. Map of sample locations in the boreal ecoregion of Alberta. Inset map shows region within Canada, with boreal ecoregion shaded.


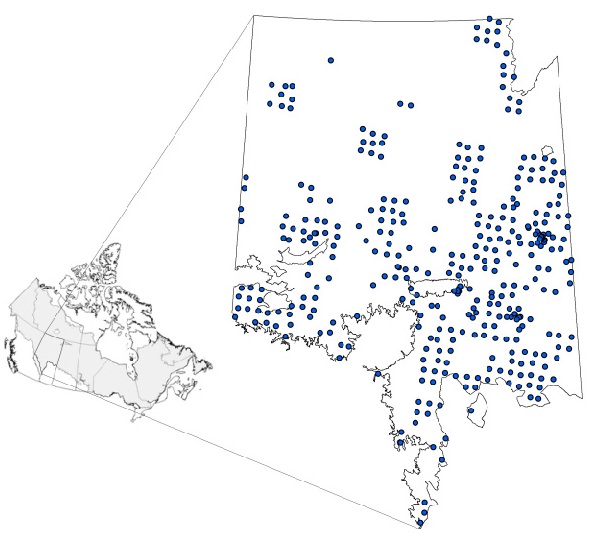

Supplement: S1 Fig — Inset map shows region within Canada, with boreal ecoregion shaded. (DOCX) [file pone.0125579.s001.docx]
