# Supplementary material for: Scaling Disturbance Instead of Richness to Better Understand Anthropogenic Impacts on Biodiversity
Source: PLoS One. 2015 May 7;10(5):e0125579. doi: 10.1371/journal.pone.0125579 (PMC4423832; doi:10.1371/journal.pone.0125579)
Supplement: S1 Table — (DOCX) [file pone.0125579.s002.docx]

Table S1. Comparison of model shapes of species richness-human disturbance relationships at local (1 ha) and landscape (18 km^2^) scales.

| **Measurement scale of disturbance** | **Model** | **Equation** | **% human disturbance at peak richness** | ***p*** | **df** | ***p* of increase in *r^2^* of quadratic over linear model** | ***r^2^*** | **AIC score** |
| --- | --- | --- | --- | --- | --- | --- | --- | --- |
| 1 ha | linear | y = 0.00068x + 3.84212 |  | 0.229 | 367 |  | 0.004 | 3296.0 |
|  | quadratic | y = -0.00018x^2^ + 0.002x + 3.789 | 50.333 | < 0.001 | 366 | < 0.001 | 0.108 | 3270.7 |
|  |  |  |  |  |  |  |  |  |
| 18 km^2^ | linear | y = -0.00300x + 3.92917 |  | < 0.001 | 367 |  | 0.027 | 3286.5 |
|  | quadratic | y = -0.00026x^2^ + 0.00161x + 3.799 | 35.05 | < 0.001 | 366 | < 0.001 | 0.122 | 3249.6 |
| 1 ha & 18km^2^ | y = -0.00016x_local_^2^ - 0.00020x_landscape_^2^ + 0.016580x_local_ + 0.01200x_landscape_ + 3.745 | | N/A | < 0.001 | 365 | N/A | 0.226 | 3224.6 |
