# Supplementary material for: Scaling Disturbance Instead of Richness to Better Understand Anthropogenic Impacts on Biodiversity
Source: PLoS One. 2015 May 7;10(5):e0125579. doi: 10.1371/journal.pone.0125579 (PMC4423832; doi:10.1371/journal.pone.0125579)
Supplement: S2 Table — All measures observed or estimated at site centre or within entire local 1 ha site, unless otherwise noted. Details are available from the Alberta Biodiversity Monitoring Institute at www.abmi.ca. (DOCX) [file pone.0125579.s003.docx]

Table S2. Explanatory variables included in this study. All measures observed or estimated at site centre or within entire local 1 ha site, unless otherwise noted. Details are available from the Alberta Biodiversity Monitoring Institute.

| **Variable** | **Description** |
| --- | --- |
| Human disturbance | Proportion of area altered by human land use as assessed with aerial and satellite imagery |
| Agricultural disturbance | Proportion of area altered by pasture and croplands |
| Forestry cut areas | Proportion of area altered by forestry activities. Variable in time since disturbance |
| Hard linear disturbance | Proportion of area altered by permanent and intense disturbance such as roads and railways |
| Soft linear disturbance | Proportion of area altered by temporary disturbances which allow for successional processes such as pipelines, powerlines, and cutlines (primarily of oil/gas explorations) |
| Urban/industrial disturbance | Proportion of area altered by non-linear permanent and intense features such as settlements coal and mineral surface mines, oil and gas well pads, communication towers, gravel pits, spoil pads, and heavy oil sands development |
| Natural subregion | Ecological classification of geographic units within the boreal ecoregion based on landscape patterns in climate, physiographic features, vegetation, soil, wildlife and land use attributes [1] |
| Latitude |  |
| Longitude |  |
| Elevation |  |
| Topographic heterogeneity | Index of topographic ruggedness, expresses spatial variation in elevation [2] |
| Growing degree days | Heat accumulation, or annual sum of mean daily temperature degrees > 5 °C |
| Mean annual temperature | Estimated by Alberta Climate Model [3] based on climatological records of Environment Canada |
| Mean annual precipitation | Estimated by Alberta Climate Model [3] based on climatological records of Environment Canada |
| Terrain wetness | Terrain based site wetness derived from digital elevation model using moisture flows and retention [4] |
| Site wetness | Observed proportion of 1 ha area in wetland |
| Solar flux | Estimated annual solar irradiance (MJ/cm^2^) [5] |
| Canopy closure | Mean amount of sky obscured by canopy using densitometer at 8 specific points in local site |
| Oldest tree age | Age of oldest tree determined by tree core/growth ring analysis |
| Organic depth | Mean depth of organic soil layer (cm) |
| Soil type | Dominant soil sub-order classification in local site |
| Surficial geology | Dominant geological classification of surficial materials in local site |
| Slope position | Index of topographic position on slope based on elevation [6] |
| Landform classification | General shape of landscape feature, such as upland drainage or local ridge [6] |

**Additional References for Table S2**

1. Natural Regions Committee (2006) Natural Regions and  Subregions of Alberta. Alberta, Canada.

2. Riley SJ, DeGloria SD, Elliot R (1999) A terrain ruggedness index that quantifies topographic heterogeneity. Intermt J Sci 5: 23–27.

3. Alberta Environment (2005) Alberta  Climate Model (ACM) to provide climate estimates (1961-1990) for any location in Alberta from its geographic coordinates. Alberta, Canada.

4. Gessler PE, Althouse L, Chamran F, Chadwick OA, Holmes K (2000) Modeling soil–landscape and ecosystem properties using terrain attributes. Soil Sci Soc Am J 64: 2046–2056.

5. McCune B (2007) Improved estimates of incident radiation and heat load using non‐parametric regression against topographic variables. J Veg Sci 18: 751–754.

6. Jenness J (2006) Topographic Position  Index (tpi_jen.avx) extension for ArcView 3.x, v. 1.2. Available at: http://www.jennessent.com/arcview/tp.
