# Supplementary material for: Scaling Disturbance Instead of Richness to Better Understand Anthropogenic Impacts on Biodiversity
Source: PLoS One. 2015 May 7;10(5):e0125579. doi: 10.1371/journal.pone.0125579 (PMC4423832; doi:10.1371/journal.pone.0125579)
Supplement: S3 Table — Numbers below the diagonal indicate the correlation. Bold values indicate adjusted p < 0.05. Numbers above the diagonal indicate the adjusted p-value. (DOCX) [file pone.0125579.s004.docx]

Table S3. Correlation matrix of human disturbance and continuous environmental covariates. Numbers below the diagonal indicate the correlation. Bold values indicate adjusted p < 0.05. Numbers above the diagonal indicate the adjusted p-value.

|  | Local human disturbance | Landscape human disturbance | Latitude | Longitude | Elevation | Topographic heterogeneity | Growing degree days | Mean annual temperature | Mean annual precipitation | Terrain wetness | Site wetness | Solar flux | Canopy closure | Organic depth |
| --- | --- | --- | --- | --- | --- | --- | --- | --- | --- | --- | --- | --- | --- | --- |
| Local human disturbance |  | 0 | 0.001 | 1 | 1 | 1 | 0.039 | 0.001 | 0.008 | 0.013 | 0 | 0.006 | 1 | 0.036 |
| Landscape human disturbance | **0.34** |  | 0 | 1 | 0.012 | 1 | 0 | 0 | 1 | 1 | 0.074 | 0 | 0.006 | 0.076 |
| Latitude | **-0.31** | **-0.53** |  | 0 | 0.391 | 0 | 0 | 0 | 0.015 | 0.013 | 0 | 1 | 1 |  |
| Longitude | 0.1 | -0.13 | -0.05 |  | 0.001 | 1 | 0.026 | 1 | 1 | 1 | 1 | 1 | 0.898 | 1 |
| Elevation | 0.15 | **0.27** | **-0.68** | **-0.31** |  | 1 | 0 | 0 | 0 | 0.003 | 0.001 | 0 | 1 | 1 |
| Topographic heterogeneity | 0.11 | -0.01 | -0.19 | -0.07 | 0.08 |  | 0.797 | 0.124 | 1 | 0 | 0 | 0.002 | 0 | 0.030 |
| Growing degree days | **0.24** | **0.36** | **-0.39** | **0.25** | **-0.34** | 0.17 |  | 0 | 1 | 1 | 1 | 0.001 | 1 | 1 |
| Mean annual temperature | **0.31** | **0.51** | **-0.93** | -0.11 | **0.53** | 0.22 | **0.56** |  | 0 | 0.220 | 0.139 | 0 | 1 | 1 |
| Mean annual precipitation | **0.27** | 0.04 | **-0.66** | 0.09 | **0.69** | 0.13 | -0.04 | **0.58** |  | 0 | 0.797 | 0 | 1 | 1 |
| Terrain wetness | **-0.27** | 0.03 | **0.26** | -0.13 | **-0.29** | **-0.47** | -0.03 | -0.2 | **-0.33** |  | 0 | 0.014 | 0.120 | 0.010 |
| Site wetness | **-0.38** | -0.23 | **0.27** | 0.06 | **-0.3** | **-0.36** | -0.03 | -0.21 | -0.17 | **0.57** |  | 0.081 | 0.276 | 0 |
| Solar flux | **0.28** | **0.38** | **-0.74** | 0.02 | **0.49** | **0.3** | **0.31** | **0.7** | **0.48** | **-0.26** | -0.23 |  | 1 | 1 |
| Canopy closure | 0.07 | **0.28** | -0.08 | 0.16 | 0.04 | **-0.35** | **-0.05** | -0.02 | 0.01 | 0.22 | 0.2 | 0.01 |  | 0 |
| Organic depth | **-0.25** | -0.23 | 0.03 | 0.06 | 0.01 | **-0.25** | **-0.1** | -0.03 | 0.14 | **0.27** | **0.37** | -0.04 | **0.35** |  |
