# Supplementary material for: Scaling Disturbance Instead of Richness to Better Understand Anthropogenic Impacts on Biodiversity
Source: PLoS One. 2015 May 7;10(5):e0125579. doi: 10.1371/journal.pone.0125579 (PMC4423832; doi:10.1371/journal.pone.0125579)
Supplement: S4 Table — The negative binomial regression models were constructed using all parameters (saturated) to facilitate comparisons across models. (DOCX) [file pone.0125579.s005.docx]

Table S4. Species richness-human disturbance relationships at local and landscape scales, accounting for co-variates. The negative binomial regression models were constructed using all parameters (saturated) to facilitate comparisons across models.

| **Measurement scale of disturbance** | **Model** | ***p* of disturbance parameter** | **df** | ***p* of increase in *r^2^* of quadratic over linear model** | ***r^2^*** | **AIC score** |
| --- | --- | --- | --- | --- | --- | --- |
| None | N/A | N/A | 192 | N/A | 0.711 | 1573.9 |
| 1 ha | linear | 0.015 | 191 |  | 0.722 | 1573.3 |
|  | quadratic | 0.032 | 190 | 0.032 | 0.730 | 1570.7 |
| 18 km^2^ | linear | 0.120 | 191 |  | 0.711 | 1573.9 |
|  | quadratic | 0.948 | 190 | 0.086 | 0.711 | 1575.9 |
| 1 ha & 18 km^2^ | N/A | 0.019, 0.647 | 189 | N/A | 0.738 | 1535.7 |
