# Supplementary material for: Scaling Disturbance Instead of Richness to Better Understand Anthropogenic Impacts on Biodiversity
Source: PLoS One. 2015 May 7;10(5):e0125579. doi: 10.1371/journal.pone.0125579 (PMC4423832; doi:10.1371/journal.pone.0125579)
Supplement: S5 Table — (DOCX) [file pone.0125579.s006.docx]

Table S5. Comparison of models of richness-disturbance relationships for native and exotic species

|  | **Measurement scale of disturbance** | **Model** | **Equation** | **% human disturbance at peak richness** | ***p*** | **df** | ***p* of increase in *r^2^* of quadratic over linear model** | ***r^2^*** | **AIC score** |
| --- | --- | --- | --- | --- | --- | --- | --- | --- | --- |
| Native species richness | 1 ha | Linear | y = -0.0009565x + 3.77993 |  | 0.168 | 367 |  | 0.005 | 3334.990 |
|  |  | quadratic | y = -0.0001695x^2^ + 0.01540x  + 3.731 | 46.141 | < 0.001 | 366 | < 0.001 | 0.077 | 3321.361 |
|  |  |  |  |  |  |  |  |  |  |
|  | 18 km^2^ | linear | y = -0.0078350x + 3.90660 |  | < 0.001 | 367 |  | 0.022 | 3289.817 |
|  |  | quadratic | y = -0.0003462x^2^ + 0.01975x  + 3.720 | 28.723 | < 0.001 | 366 | < 0.001 | 0.113 | 3230.435 |
|  |  |  |  |  |  |  |  |  |  |
| Exotic species richness | 1 ha | linear | y = 0.019917x + 0.23262 |  | < 0.001 | 367 |  | < 0.001 | 1532.476 |
|  |  | quadratic | y = 0.0005026x^2^ + 0.068829x  + 0.013199 | no peak | < 0.001 | 366 | < 0.001 | 0.047 | 1507.403 |
|  |  |  |  |  |  |  |  |  |  |
|  | 18 km^2^ | linear | y = -0.028812x + 0.190554 |  | < 0.001 | 367 |  | 0.026 | 1515.433 |
|  |  | quadratic | y = -0.0003676x^2^ + 0.0600983x  -0.0624855 | no peak | < 0.001 | 366 | 0.001 | 0.067 | 1507.307 |
