# Supplementary material for: Scaling Disturbance Instead of Richness to Better Understand Anthropogenic Impacts on Biodiversity
Source: PLoS One. 2015 May 7;10(5):e0125579. doi: 10.1371/journal.pone.0125579 (PMC4423832; doi:10.1371/journal.pone.0125579)
Supplement: S6 Table — Only the disturbance types and forms selected by the model are shown. Linear and quadratic forms of each disturbance variable were options, and quadratic variables are indicated by “2”. (DOCX) [file pone.0125579.s007.docx]

Table S6. Best models (stepwise selected) explaining richness with types of human disturbance at each scale individually. Only the disturbance types and forms selected by the model are shown. Linear and quadratic forms of each disturbance variable were options, and quadratic variables are indicated by “^2^”.

| **Measurement scale of disturbance** | **Human disturbance type** | **Estimate** | **df** | ***r^2^*** | ***p*** | **AIC** |
| --- | --- | --- | --- | --- | --- | --- |
| 1 ha | Forestry | 0.002319 | 363 | 0.269 | < 0.001 | 3198.41 |
|  | Soft linear features | 0.007410 |  |  | 0.003 |  |
|  | Urban and industrial | 0.005663 |  |  | < 0.001 |  |
|  | Agriculture^2^ | -0.0001646 |  |  | < 0.001 |  |
| 18 km^2^ | Hard linear features | 0.09235 | 366 | 0.128 | < 0.001 | 3253.50 |
|  | Agriculture^2^ | -0.0001005 |  |  | < 0.001 |  |
