# Supplementary material for: Scaling Disturbance Instead of Richness to Better Understand Anthropogenic Impacts on Biodiversity
Source: PLoS One. 2015 May 7;10(5):e0125579. doi: 10.1371/journal.pone.0125579 (PMC4423832; doi:10.1371/journal.pone.0125579)
Supplement: S7 Table — Only the disturbance types, their scales, and the variable forms selected by the model are shown. Linear and quadratic forms of each disturbance variable at each scale were options, and quadratic variables are indicated by “2”. (DOCX) [file pone.0125579.s008.docx]

Table S7. Best multi-scale model of richness explained by human disturbance types at local and landscape scales. Only the disturbance types, their scales, and the variable forms selected by the model are shown. Linear and quadratic forms of each disturbance variable at each scale were options, and quadratic variables are indicated by “^2^”.

| **Human disturbance type and scale** | **Estimate** | **df** | ***r^2^*** | ***p*** | **AIC** |
| --- | --- | --- | --- | --- | --- |
| Forestry (1ha) | 0.002271 | 358 | 0.307 | < 0.001 | 3192.3 |
| Hard linear features (1 ha) | 0.004651 |  |  | 0.128 |  |
| Soft linear features (1 ha) | 0.006840 |  |  | 0.004 |  |
| Urban and industrial (1 ha) | 0.005203 |  |  | < 0.001 |  |
| Agriculture^2^ (1 ha) | -0.0001887 |  |  | 0.002 |  |
| Hard linear features (18 km^2^) | 0.05066 |  |  | 0.006 |  |
| Agriculture^2^ (18 km^2^) | -0.0001106 |  |  | 0.021 |  |
